# An idiopathic severe macroglossia in a young adult patient: a rare case

**DOI:** 10.1093/jscr/rjae313

**Published:** 2024-05-18

**Authors:** Bassem Al Hariri, Muad Abdi Hassan, Muhammad Sharif, Rawan S Mohamed, Hadil Altaj Altrify Alsidig, Hind Altag Alteraify Alsiddig, Memon Noor Illahi

**Affiliations:** Department of Medicine, Hamad Medical Corporation, Doha, Qatar; Weill Cornell Medicine-Qatar, Doha, Qatar; Medical Education Department, Hamad Medical Corporation, Doha, Qatar; Department of Medicine, Hamad Medical Corporation, Doha, Qatar; Medical Education Department, Hamad Medical Corporation, Doha, Qatar; Medical Education Department, Hamad Medical Corporation, Doha, Qatar; Medical Education Department, Hamad Medical Corporation, Doha, Qatar; Department of Medicine, Hamad Medical Corporation, Doha, Qatar

**Keywords:** macroglossia, idiopathic, isolated macroglossia, rapid progression

## Abstract

Macroglossia, an uncommon anatomical anomaly, can manifest as either congenital or acquired. The size of the tongue undergoes variations with age, peaking at 8 years and reaching full maturity at 18 years. Congenital macroglossia stems from diverse conditions, such as muscular hypertrophy, hemangioma, lymphangioma, Down syndrome, and others. Acquired macroglossia can result from malignancies, endocrine and metabolic disorders, chronic infectious diseases, and head and neck infections, among other factors. Additionally, extended-prone surgery can lead to its development. The incidence of macroglossia is likely underreported. This presentation is rare with only six reported cases in the literature.

## Introduction

The normal size of the tongue varies with age, reaching its maximum in the first 8 years and attaining full maturity at 18 [1]. Macroglossia, an uncommon anatomical anomaly, typically indicates an underlying condition and rarely occurs in isolation. Isolated macroglossia is exceptionally rare and inherited as an autosomal dominant trait [2]. The etiology of macroglossia is multifaceted and classified into acquired and congenital conditions. Major causes of congenital macroglossia include idiopathic muscular hypertrophy, adenoid hyperplasia, hemangioma, lymphangioma, Down syndrome, and various syndromes and diseases. Acquired disorders are often associated with malignancies, endocrine and metabolic disorders, chronic infectious diseases, and trauma, among others [2]. There are documented cases of macroglossia developing postextended prone surgery, emphasizing the need for proper precautions [3]. The estimated incidence of macroglossia in posterior fossa surgery in a sitting position is ~1% [4]. Despite this, the true incidence of macroglossia is unknown, and there is a prevailing belief that it is underreported. The CARE Checklist has been completed by the authors for this case report, attached as online Supplementary Material 1.

## Case presentation

**Figure 1:**
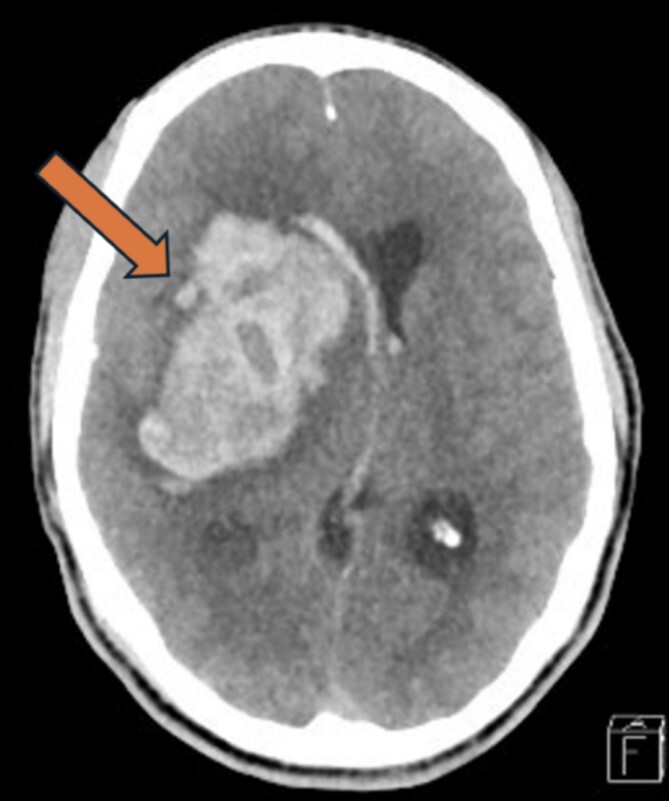
Head CT-Scan showing large intraparenchymal hematoma involving the right basal ganglia and temporal lobe with surrounding edema, extending into the lateral, third, and fourth ventricles, causing a midline shift of 13 mm with effacement of the right lateral ventricle and adjacent sulci.

A 30-year-old male was known to be hypertensive and not compliant with his medication. He was in the mosque with his friend when he developed a sudden episode of headache, and his friend called the ambulance. When he arrived at the emergency department (ED), facial droop was observed with confusion, and he was moving his left side less than the right. There was no vomiting or seizure; vitally his blood pressure was 200/190, his respiratory rate was 18 breaths/min, and pulse was 82 bpm. His Glasgow Coma Scale (GCS) is 3/15. The patient was intubated, and an urgent CT scan of the head showed a large intraparenchymal hematoma involving the right basal ganglia and temporal lobe with surrounding edema, extending into the lateral, third, and fourth ventricles, causing a midline shift of 13 mm with effacement of the right lateral ventricle and adjacent sulci (Fig. 1).

**Figure 2:**
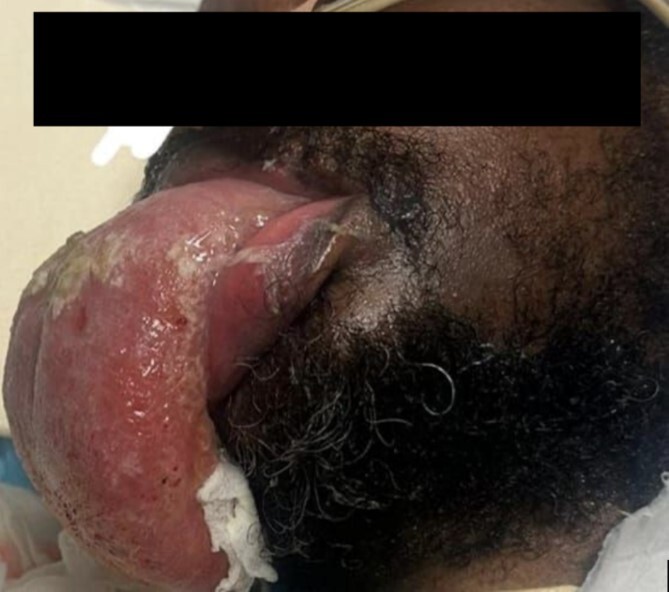
Enlarged tongue.

**Figure 3:**
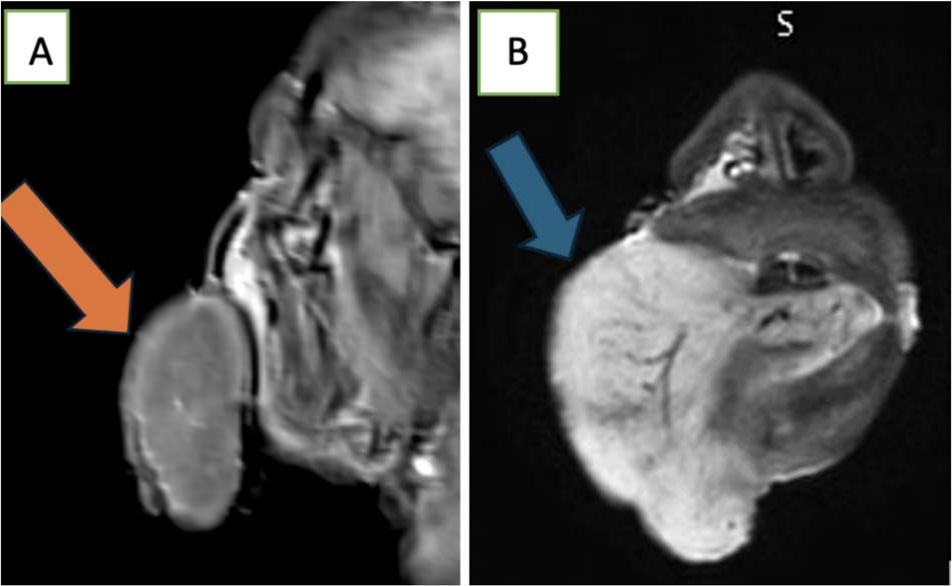
MRI Tongue shows significant macroglossia as seen in the (A) and (B) above with significant vascular engorgement these MRI features along with its subacute evolution are suggestive of being angioneurotic.

**Figure 4:**
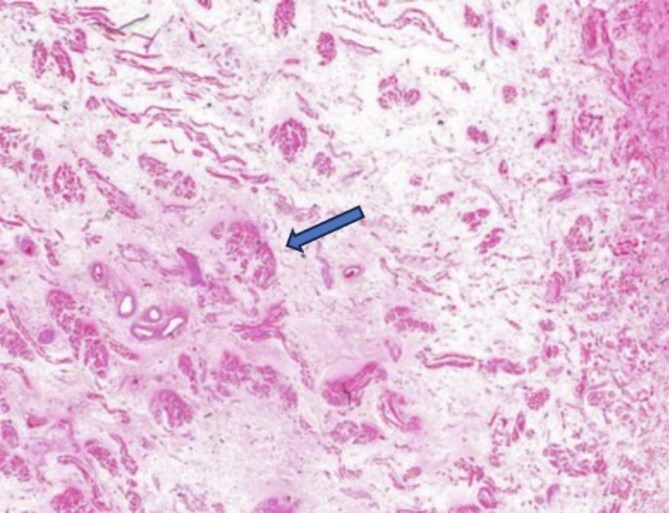
A microscopic study of the patient’s tongue tissue revealed chronic inflammation, fat infiltration in the submucosa, and striated muscle degeneration.

Left-side frontal external ventricular drainage (EVD) insertion was done, followed by right-side craniotomy, and evacuation of intracranial hematoma was done for him with adequate control of his blood pressure. Thirty-one days after the operation, the patient experienced significant swelling of the tongue, as shown in Fig. 2. The doctors suspected an allergic reaction and started the patient on steroids and diphenhydramine. However, despite stopping all medications that could have caused an allergic reaction, the swelling persisted and worsened. He underwent an MRI of the tongue, which showed significant macroglossia above with significant vascular engorgement (Fig. 3). Lab results showed a mild increase in C1 esterase inhibitor (Table 1). He underwent tongue-reduction surgery to alleviate his symptoms. Microscopic study of the patient’s tongue tissue revealed chronic inflammation and fat infiltration in the submucosa, along with striated muscle degeneration. There was no evidence of dysplasia or malignancy, and tests for amyloidosis were negative (Fig. 4).

**Table 1:** Immunology work-up.

**Laboratory result**	**Values**	**Normal values**
C4	0.33gm/L	0.10–0.40gm/L
C1 esterase inhibitor	0.55gm/L	0.21–0.38gm/L

## Discussion

Macroglossia following surgical interventions, especially neurosurgical procedures, poses a complex challenge in terms of both diagnosis and management. While the presented case highlights the occurrence of macroglossia postintracranial surgery, the underlying mechanisms and contributing factors remain elusive. The literature suggests various potential causes, including regional vascular and lymphatic complications, reperfusion injury, neurological factors, and trauma-related conditions [5].

The incidence of postoperative macroglossia, particularly in neurosurgical cases, is not well-documented, leading to a lack of understanding of its true prevalence and contributing factors. The rarity of this condition, combined with its potential for adverse outcomes, such as airway obstruction and prolonged ICU stays, emphasizes the need for increased awareness and research in this area [5].

In the presented case, the patient’s history of hypertension and non-compliance with medication introduces additional complexities. The initial manifestation of significant intracranial hemorrhage necessitated urgent surgical intervention, leading to a successful craniotomy and hematoma evacuation. However, the subsequent development of macroglossia raised questions about the potential links between the surgical procedure and tongue swelling.

The differential diagnosis process in this case was comprehensive, involving the exclusion of allergic reactions, consideration of immunological factors (mild increase in C1 esterase inhibitor), and ruling out malignancies or amyloidosis. Despite these efforts, the precise cause of macroglossia remained elusive. The decision to perform a tongue reduction surgery was made after excluding all other potential causes, emphasizing the importance of a thorough diagnostic approach.

The literature review suggests that macroglossia can lead to significant morbidity, affecting various aspects of a patient’s life, including speech, swallowing, and airway patency. In this case, the prolonged ICU stay and the subsequent development of macroglossia highlight the potential complications associated with postoperative recovery [6].

Furthermore, the discussion delves into the challenges of managing macroglossia, particularly when it is unresponsive to conventional treatments. The interdisciplinary collaboration between anesthesiologists and surgeons is emphasized as critical in optimizing preventative measures. The importance of avoiding excessive neck flexion or rotation and promoting venous drainage during surgery is underscored to mitigate the risk of macroglossia development. In conclusion, the presented case sheds light on the intricacies of diagnosing and managing postoperative macroglossia, particularly in the context of neurosurgical interventions. Further research is warranted to better understand the incidence, risk factors, and optimal management strategies for this rare but potentially serious complication. The joint efforts of healthcare professionals are crucial in ensuring a comprehensive and timely approach to mitigate the impact of macroglossia on patient outcomes.

## Conclusion

Macroglossia is a rare condition characterized by an enlarged tongue. This case underscores the importance of considering macroglossia in postoperative complications, especially in neurosurgical procedures. Thorough assessment, prompt identification of potential causes, and timely interventions are crucial for managing macroglossia and minimizing associated morbidity. The joint efforts of anesthesiologists and surgeons are pivotal in implementing preventive measures and optimizing patient outcomes.

## Data Availability

The data that support the findings of this study are available in this article.
